# Tetracaine hydrochloride gel delays and reduces catheter-related bladder discomfort after transurethral resection: a randomized controlled trial

**DOI:** 10.7717/peerj.21891

**Published:** 2026-11-02

**Authors:** Yang Liu, Ling Li, Jingjin Li, Feng Feng, Haoran Wang, Zhenfeng Zhang, Zijian Zhao, Qingchun Sun

**Affiliations:** 1 Department of Anesthesiology and Perioperative Medicine, The First Affiliated Hospital with Nanjing Medical University, Nanjing, Jiangsu, China; 2 Department of Anesthesiology, Chongqing Hospital of Jiangsu Province Hospital, Chongqing, China

**Keywords:** Tetracaine, Transurethral resection of prostate, Transurethral resection of bladder, Adverse effects, Randomized controlled trial, Anesthetics, local, Catheters, indwelling

## Abstract

**Background:**

Catheter-related bladder discomfort (CRBD) results from involuntary bladder contractions triggered by both the indwelling urinary catheter and surgical trauma during transurethral resection. Systemic analgesics and anticholinergics are often used but carry risks of side effects. Topical anesthetics like tetracaine hydrochloride gel offer a potential targeted approach, but their efficacy in delaying and reducing CRBD remains unclear.

**Methods:**

In this randomized, single-blind study, patients undergoing transurethral resection were allocated to the tetracaine gel group (*n* = 65) or the lubricant control group (*n* = 65). Before catheter insertion, three g of tetracaine gel or standard lubricant was evenly applied to the catheter surface. The primary outcome was the time to first-reported moderate-to-severe CRBD after tracheal extubation. Secondary outcomes included the incidence of new-onset moderate and severe CRBD at specified intervals, as well as the patient satisfaction, postoperative pain scores, analgesic requirements, and adverse events. The incidence of moderate-to-severe CRBD stratified by age and procedure type was also analyzed.

**Results:**

The time to first-reported moderate-to-severe CRBD was significantly longer in the tetracaine gel group compared to the control group (30 [10.0–61.3] min *vs.* 5 [4.8–10.0] min, *p* < 0.001) among patients who developed this event. The incidence of new-onset moderate-to-severe CRBD was significantly lower in the tetracaine gel group within 30 min (15 [23.1%] *vs.* 43 [66.2%]; RR = 0.153, 95% CI [0.071–0.332]; *p* < 0.001) and over the total 24 hours assessment period (30 [46.2%] *vs.* 46 [70.8%]; RR = 0.354, 95% CI [0.172–0.730]; *p* = 0.004). A transient increase in incidence was noted in the tetracaine group between 30–120 min (11 [16.9%] *vs.* 2 [3.1%]; RR = 6.417, 95% CI [1.362–30.228]; *p* = 0.016). The non-significant interaction test indicates that the effect magnitude of tetracaine gel did not differ by age (*P* for interaction = 0.392) or procedure type (*P* for interaction = 0.883). Patient satisfaction at 6 hours was higher (6.7 ± 0.5 *vs.* 6.2 ± 0.8, *p* < 0.001), and postoperative pain scores were lower at all assessed time points. No significant differences were found in analgesic requirements, postoperative delirium, infections, or hospital stay.

**Conclusions:**

Topical application of tetracaine gel to the urinary catheter effectively delays the onset and reduces the incidence of moderate-to-severe CRBD in the early postoperative period.

## Introduction

Transurethral resection of bladder tumor (TURBT) and transurethral resection of the prostate (TURP) are common urological procedures that typically require postoperative urinary catheterization (Souiden et al., 2025). Upon emergence from general anesthesia, catheterized patients may experience an urge to void or discomfort in the suprapubic region, a condition termed catheter-related bladder discomfort (CRBD). Symptoms resembling an overactive bladder, such as urgency, frequency, and restlessness, can significantly contribute to emergence agitation in the post-anesthesia care unit (PACU) (Xu & Sun, 2026). The reported incidence of CRBD is high, ranging from 47% to 90%, and symptoms typically occur within the first few hours after surgery (Abo Zeid et al., 2026; Veerman et al., 2021; Wilde et al., 2017). Notably, its occurrence is approximately three times higher in male patients, likely due to their longer and more complex urethral anatomy. CRBD can provoke agitation, hypertension, and tachycardia, potentially impairing postoperative recovery (Abo Zeid et al., 2026).

Current management of CRBD relies primarily on systemic analgesics or anticholinergic agents (Wang et al., 2025; Park et al., 2024; Wang et al., 2026). These approaches, however, are often limited by systemic side effects such as respiratory depression, nausea, vomiting, dry mouth, and cardiac irregularities, particularly in elderly patients or those with comorbidities. Consequently, investigating targeted local anesthetic strategies to inhibit nociceptive transmission directly from the urethral and bladder mucosa has gained interest. Topical anesthetic application offers a theoretical basis for mitigating CRBD by providing surface anesthesia to the urethral mucosa, thereby attenuating afferent painful stimuli. An *in vivo* animal study demonstrated that a lidocaine-loaded carrier incorporated into a standard urinary catheter could achieve sustained lidocaine release, thereby alleviating CRBD (Kim et al., 2021). Currently available topical anesthetics for urethral catheterization include lidocaine gel, prilocaine-lidocaine cream, and benzocaine spray, with gel formulations preferred for uniform mucosal coating.

Tetracaine hydrochloride gel, an ester-type local anesthetic, blocks sodium channels to inhibit neuronal conduction. Its gel formulation prolongs analgesic action, making it well-suited for skin and mucosal application (Zhao et al., 2024; Schivone et al., 2019). To our knowledge, no published animal or clinical studies have specifically evaluated tetracaine gel for CRBD prevention. Whether tetracaine gel can delay the onset and reduce the incidence of this complication remains unclarified. Therefore, this study aimed to evaluate the efficacy of catheter-coated tetracaine gel in male patients undergoing TURBT or TURP. We hypothesized that tetracaine gel would prolong the time to first-reported moderate-to-severe CRBD and reduce its occurrence compared to standard lubricant.

## Materials & Methods

This prospective, randomized, single-blind, controlled trial was conducted at the First Affiliated Hospital with Nanjing Medical University (Jiangsu Province Hospital) in Nanjing, China. The study protocol was approved by the Ethics Committee of the First Affiliated Hospital with Nanjing Medical University (no. 2025-SR-534) and then was registered with the Chinese Clinical Trial Registry on July 31, 2025 (https://www.chictr.org.cn/indexEN.html, ChiCTR2500106854). Written informed consent was obtained from all participants prior to their enrollment. The trial was conducted in accordance with the approved protocol and the principles of the Declaration of Helsinki.

### Study population

Male patients scheduled for elective TURBT or TURP between August 2025 and December 2025 were assessed for eligibility. We initially screened 158 patients, among whom 130 were randomized and included in the final analysis. The inclusion criteria were: age >18 years; American Society of Anesthesiologists (ASA) Physical Status I to III; and scheduled for elective TURBT or TURP. The exclusion criteria were: a history of poorly controlled hypertension or severe cardiocerebrovascular disease; severe hepatic or renal dysfunction; presence of psychiatric disorders or inability to communicate and cooperate; a known allergy to local anesthetics; and refusal to provide written informed consent.

### Randomization, concealment, and blinding

Eligible participants were randomly divided into two groups (65 participants per group): the tetracaine gel group and the lubricant (control) group. Randomization was performed using an online randomization program (Random Allocation Software version 1.0; Isfahan University of Medical Sciences, Isfahan, Iran) with block sizes of four and an allocation ratio of 1:1, according to a computer-generated randomization schedule prepared before the initiation of patient recruitment. Participants were blinded to treatment allocation.

The randomization codes were secured in sequentially numbered, identical, opaque, and sealed envelopes, which were kept in a locked box throughout the study. The allocation sequence was concealed by the first investigator. Prior to catheter insertion, the first investigator, who was not blinded to group assignment, provided the surgeon with either tetracaine gel or lubricant according to the allocation code. Surgeons were not blinded due to visible differences between tetracaine gel and lubricant; however, they did not participate in any outcome assessment. Anesthesia induction and maintenance were managed by the second investigator, who was blinded to group allocation. Outcome assessments in the PACU and the general ward were performed by the third investigator, who was also blinded. To minimize bias, the pre-determined randomization order was strictly followed for all consecutive participants. Compliance with the allocation sequence was verified by an independent investigator upon completion of data collection.

### Anesthesia and monitoring

On the day before surgery, patients were educated on how to recognize symptoms of CRBD, such as suprapubic discomfort or a burning sensation accompanied by an urge to void, and how to differentiate them from surgical pain.

Upon arrival at the operating room, patients were monitored according to our institutional standards. Anesthesia was induced with 0.05 mg/kg of midazolam, two mg/kg of 1% propofol or 0.3 mg/kg of etomidate, 0.6 mg/kg of rocuronium, and 0.5 µg/kg of sufentanil. Following tracheal intubation, anesthesia was maintained with one vol% sevoflurane, a continuous infusion of propofol (4–6 mg/kg/h), remifentanil (0.3 µg/kg/min), and rocuronium (5–10 µg/kg/h). The depth of anesthesia was monitored using the bispectral index (BIS), which was maintained between 40 and 60. The administration of sevoflurane was intermittently adjusted according to the BIS values and hemodynamic parameters.

At the conclusion of the surgical procedure, the surgeon inserted a triple-lumen urethral catheter (typically 22–24 French). The catheter was lubricated with either tetracaine gel or lubricant as per randomization, its balloon inflated with 10 mL of distilled water, and continuous bladder irrigation with normal saline was commenced. Catheter insertion, gel or lubricant application, balloon inflation, irrigation, and postoperative catheter care were all carried out following standardized procedures. The patient was then transferred to the PACU for recovery. Residual neuromuscular blockade was antagonized with sugammadex. Tracheal extubation was performed after recovery of consciousness (BIS > 90) and neuromuscular function.

### Study protocol

The dose of tetracaine gel was determined based on a previous study  (Mu et al., 2017). In the tetracaine gel group, the surgeon applied three g of tetracaine gel (tetracaine hydrochloride gel, specification: 1.5 g: 70 mg, product batch number: 20250810; Zhenao Jinyinhua Pharmaceutical Co., Ltd.) evenly over the entire length where the catheter could be inserted, and observed the standard waiting time of 2 min prior to insertion. Additionally, no extra gel was applied to the urethra. In the control group, an equivalent amount of standard lubricant was applied in the same manner. The standard lubricant is the medical-grade synthetic silicone oil that comes with the urinary catheter kit. It is produced by Guangzhou Well Lead Medical Co., Ltd. and does not contain local anesthetic agents or other active ingredients.

The latex urinary catheter is produced by Guangzhou Well Lead Medical Co., Ltd. Catheter size was selected at the discretion of the surgeon based on patient sex and urethral caliber. Since the patients did not have obvious urethral stenosis, a 24Fr urinary catheter was ultimately chosen for all of them. After standard sterile preparation and application of gel or lubricant, the catheter was inserted under aseptic conditions. The catheter was advanced until urine return was observed, and then advanced a further 2–3 cm before balloon inflation. The balloon was positioned in the bladder, confirmed by clinical judgment, and inflated with 10 mL of sterile water. Gentle traction was applied after balloon inflation to ensure the balloon was seated at the bladder neck, and then the catheter was released.

The speed of bladder irrigation should be adjusted according to the color of the drainage fluid. If the drainage fluid is bright red or dark red, the irrigation speed should be increased (generally 80–100 drops per minute) to prevent blood clots from forming; when the color becomes lighter, the speed can be gradually reduced (40–60 drops per minute). The irrigation protocols for TURP and TURBT are the same.

In the PACU, rescue medication was available: sufentanil (0.1 µg/kg) intravenous injection for incisional pain (Numeric Rating Scale, NRS ≥ 4), and 50 mg tramadol intravenous injection for moderate or severe CRBD. Reevaluate the severity of CRBD 30 min later. For patients with both conditions (moderate-to-severe CRBD and NRS ≥ 4), the medication (tramadol or sufentanil) was chosen based on the primary complaint, followed by reassessment. After transfer to the general ward, the analgesic protocol employing flurbiprofen axetil and dezocine was followed for pain management.

### Assessments

A variety of patient baseline characteristics were evaluated one day before surgery, including age, body mass index, ASA Physical Status, and several comorbidities, such as hypertension, diabetes mellitus, coronary artery disease, and cerebrovascular disease. Anesthetic variables included the time to tracheal extubation (interval from surgery end to extubation), operation duration, the volume of crystalloid administered and the dose of intraoperative sufentanil. Assessed surgical variables encompassed urethral stricture, tumor size, and the time to urinary catheter removal postoperatively.

The assessment for the first episode of moderate-to-severe CRBD was performed immediately after tracheal extubation in the PACU, and subsequently at predefined time points (0.5, 2, 4, 6, 12, and 24 h) after extubation. During the intervals between the two adjacent time points, patients could report their symptoms spontaneously. The time to first-reported moderate-to-severe CRBD, defined as the interval from tracheal extubation to the first occurrence of moderate-to-severe CRBD, was recorded, with each patient contributing at most one time point. All follow-up data was recorded by the trained research nurse in the case report form, who received unified training on the CRBD grading criteria. CRBD severity was graded as follows: “none” (no CRBD reported upon inquiry), “mild” (reported only upon questioning), “moderate” (spontaneously reported without behavioral response), or “severe” (spontaneously reported with accompanying behavioral responses such as attempts to remove the catheter, strong vocal reactions, or flailing limbs).

At 6 h after extubation, patient satisfaction was assessed using a seven-point Likert scale ranging from 1 (“strongly dissatisfied”) to 7 (“extremely satisfied”). Surgical site pain intensity was evaluated using a 0–10 numeric rating scale (0 = no pain, 10 = worst imaginable pain) at 0.5, 2, 4 and 6 h. Analgesic requirements within the first 24 postoperative hours, including opioids, tramadol, and non-steroidal anti-inflammatory drugs, were recorded. Postoperative delirium was assessed within 24 h after surgery using the Confusion Assessment Method, identifying acute changes in mental status, inattention, disorganized thinking, or altered consciousness. The length of hospital stay was calculated from the preoperative admission date to the date of discharge. Indicators of postoperative infection, including fever, elevated inflammatory markers, and antibiotic use, were evaluated in the general ward.

### Primary and secondary outcomes

The primary outcome was the time to first-reported moderate-to-severe CRBD among patients who developed this event. Secondary outcomes included the Kaplan–Meier analysis of time-to-event; the incidence of new-onset moderate and severe CRBD within 0–0.5, 0.5–2, 2–4, 4–6, 6–12 and 12–24 h after extubation; the incidence of moderate-to-severe CRBD stratified by age (≥65 years *vs.* <65 years) and procedure type (TURBT *vs.* TURP) within 6 h following extubation; patient satisfaction at 6 h; postoperative pain scores; postoperative analgesic requirements; postoperative delirium; postoperative infection and the duration of hospitalization.

### Statistical analysis

Based on pilot study data and previous research findings, the mean time to first report of moderate or severe CRBD was 12 min (SD 3) in patients not receiving tetracaine gel. We postulated that the application of tetracaine gel would prolong this time to a mean of 120 min (SD 30). Given 80% power and a 5% significance level, the calculated sample size was 124 patients (62 per group) using a 1:1 allocation ratio. To account for potential attrition, although the anticipated dropout rate was low, we enrolled a final sample of 130 patients (65 per group).

The primary outcome analysis was restricted to patients who developed moderate-to-severe CRBD within 24 h after tracheal extubation. If no moderate-to-severe CRBD occurred until 24 h, the patient was recorded as having no event and was not included in the primary outcome analysis. The intention-to-treat population including all randomized patients was used for secondary outcomes. Data were presented as means with standard deviations, medians with interquartile ranges, or number with percentages. The normal distribution of continuous variables was assessed using the Kolmogorov–Smirnov test. Continuous variables were compared using the independent *t*-test or the Mann–Whitney *U* test, as appropriate. Categorical variables were analyzed using the chi-square test or Fisher’s exact test.

The log-rank (Mantel-Cox) test was performed to compare the proportion of patients free from moderate-to-severe CRBD between groups and plotted the Kaplan–Meier curve. For the time-to-event analysis, participants who had not developed moderate-to-severe CRBD by 360 minutes were censored at the 360-minute time point. Once a participant experienced the first occurrence of moderate-to-severe CRBD, the participant was no longer included in the risk set for subsequent intervals. *post-hoc* subgroup analyses stratified by age and procedure type were performed using multivariable logistic regression models. Multivariable logistic regression models were adjusted for age and procedure type. To test for effect-modification, interaction terms were generated as the product of treatment-group assignment and each subgroup variable (age category and procedure type), and each interaction term was fitted separately within the adjusted model. These subgroup analyses were *post-hoc* exploratory analyses and were not prespecified in the original study protocol; no sample-size calculation was performed for these subgroup comparisons. In all analyses, a two-sided *p*-value < 0.05 was considered statistically significant. All statistical analyses were performed using SPSS version 21.0 (IBM Corp.), GraphPad Prism 10 (GraphPad Software, San Diego, CA, USA), and R version 4.3.3 (R Core Team, 2024).

## Results

The study flowchart is presented in Fig. 1. A total of 158 patients scheduled for TURBT or TURP were assessed for eligibility. Of these, 28 patients were excluded for various reasons. All 130 randomized patients were included in the final analysis, with no loss to follow-up in either group. The preoperative and intraoperative characteristics of the patients are shown in Table 1, which includes both baseline demographics and intraoperative variables. No significant differences were observed between the tetracaine gel and control groups regarding age, body mass index, ASA physical status, comorbidities, time to extubation, operation duration, volume of crystalloid administered, or tumor characteristics (type and size).

**Figure 1:**
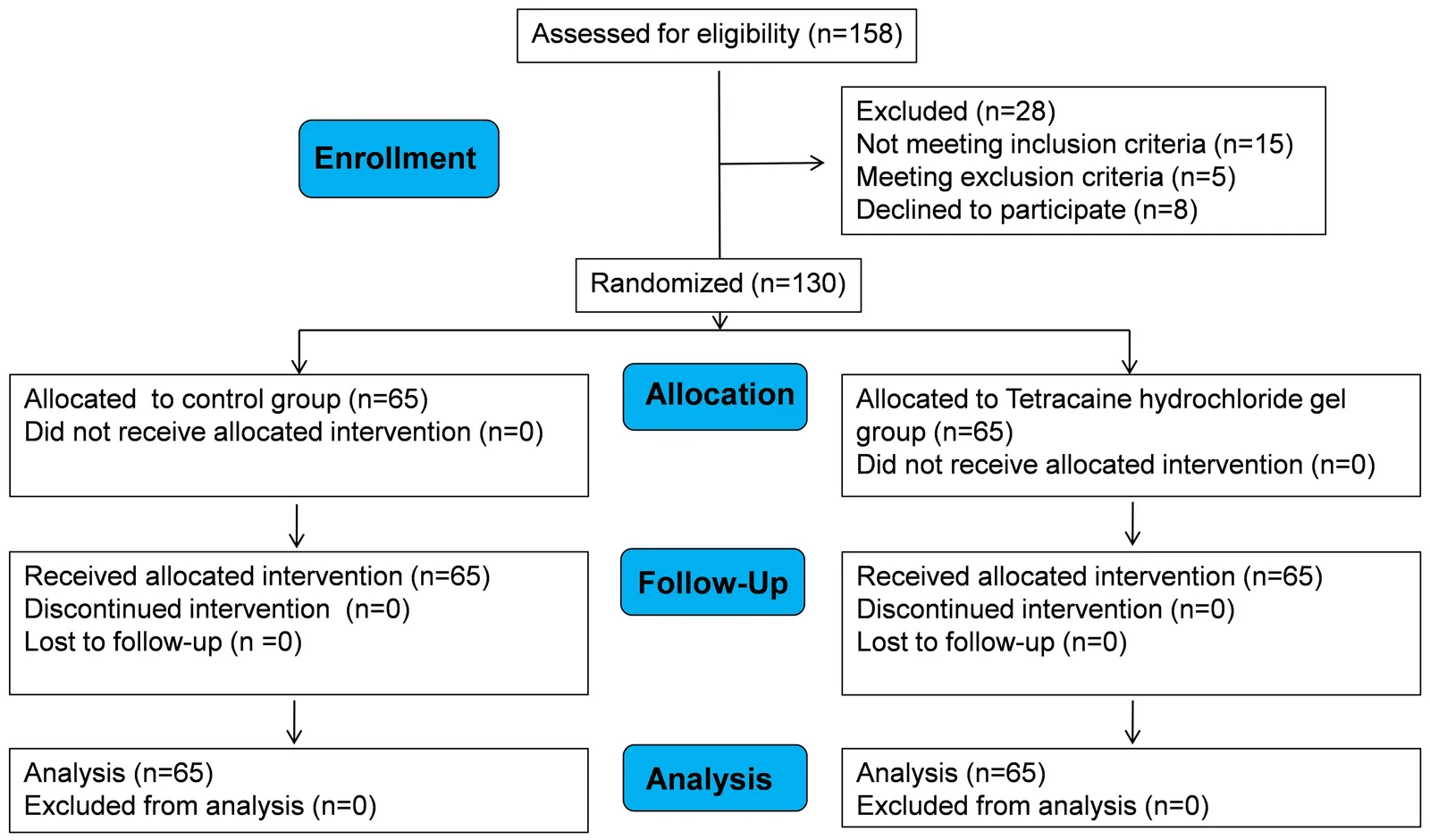
Study flowchart.

**Table 1:** Patient characteristics.

	Control group (*n* = 65)	Tetracaine gel group (*n* = 65)	*p*-value
Age, years	69 ± 8	68 ± 8	0.625
BMI, kg/m^2^	23.9 ± 2.8	24.2 ± 3.0	0.590
ASA			0.093
Class II	39 (60.0%)	48 (73.8%)	
Class III	26 (40.0%)	17 (26.2%)	
Smoking status			0.249
Never smoker	37 (56.9%)	43 (66.2%)	
Former smoker	19 (29.2%)	11 (16.9%)	
Current smoker	9 (13.8%)	11 (16.9%)	
Alcohol Consumption			0.319
Never drinker	42 (64.6%)	38 (58.5%)	
Occasional drinker	23 (35.4%)	25 (38.5%)	
Heavy Drinker	0 (0.0%)	2 (3.1%)	
Hypertension	30 (46.2%)	30 (46.2%)	0.999
Diabetes mellitus	11 (16.9%)	10 (15.4%)	0.812
Coronary artery disease	7 (10.8%)	6 (9.2%)	0.770
Cerebrovascular disease	9 (13.8%)	5 (7.7%)	0.258
Transurethral resection of the prostate	54 (83.1%)	53 (81.5%)	0.818
Transurethral resection of bladder tumor	11 (16.9%)	12 (18.5%)	0.818
Maximal prostate diameter, mm	56.0 [52.7–61.2]	56.0 [47.5–63.0]	0.238
Maximal tumor diameter, mm	16.0[12.0–23.0]	20.5[10.7–30.0]	0.621
Total intraoperative sufentanil, μg	34.2 ± 6.6	33.3 ± 6.9	0.923
Operation duration, min	60 ± 30	55 ± 34	0.464
The time to tracheal extubation, min	29 ± 12	30 ± 13	0.391
Amount of crystalloid, ml	500 [500–1,000]	500 [500–1,000]	0.528

**Notes.**

Data are expressed as means ± standard deviation, medians [interquartile range], or number (%).

ASA American Society of Anesthesiologists BMI Body mass index

All first episodes of moderate-to-severe CRBD occurred within 6 h after extubation, and no new events were identified between 6–24 h (Table 2). The time to first-reported moderate-to-severe CRBD was significantly later in the tetracaine gel group than in the control group among patients who developed this event (30 [10.0–61.3] min *vs.* 5 [4.8–10.0] min, *p* < 0.001) (Table 2). Kaplan–Meier analysis demonstrated that the time to first-reported moderate-to-severe CRBD was significantly shorter in the control group than in the tetracaine gel group. Consistently, when the tetracaine gel group was used as the reference, the control group had a higher hazard of developing moderate-to-severe CRBD (HR for control *vs.* tetracaine gel, 2.299; 95% CI [1.453–3.637]; log-rank *χ*^2^ = 14.62; *p* < 0.001) (Fig. 2).

**Table 2:** Comparison of time to first-reported moderate-to-severe CRBD and incidence of new-onset moderate and severe CRBD following extubation.

		Control group	Tetracaine gel group	*p*-value
Time to first-reported moderate-to-severe CRBD, min		5 [4.8–10.0]	30 [10.0–61.3]	<0.001
Moderate-to-severe CRBD within 24 h		46/65 (70.8%)	30/65 (46.2%)	0.004
0-0.5 h after extubation	Moderate	31/65 (47.7%)	13/65 (20.0%)	<0.001
	Severe	12/65 (18.5%)	2/65 (3.1%)	0.005
0.5–2 h after extubation	Moderate	2/65 (3.1%)	11/65 (16.9%)	0.016
	Severe	0 (0.0%)	0 (0.0%)	–
2–4 h after extubation	Moderate	1/65 (1.5%)	3/65 (4.6%)	0.619
	Severe	0 (0.0%)	0 (0.0%)	–
4–6 h after extubation	Moderate	0 (0.0%)	1/65 (1.5%)	0.999
	Severe	0 (0.0%)	0 (0.0%)	–
6–12 h after extubation	Moderate	0 (0.0%)	0 (0.0%)	–
	Severe	0 (0.0%)	0 (0.0%)	–
12–24 h after extubation	Moderate	0 (0.0%)	0 (0.0%)	–
	Severe	0 (0.0%)	0 (0.0%)	–

**Notes.**

Data are expressed as medians [interquartile range], or *n*/N (%).

Time to first-reported moderate-to-severe CRBD, defined as the interval from tracheal extubation to the first occurrence of moderate-to-severe CRBD among patients who developed this event.

CRBD Catheter-related bladder discomfort

**Figure 2:**
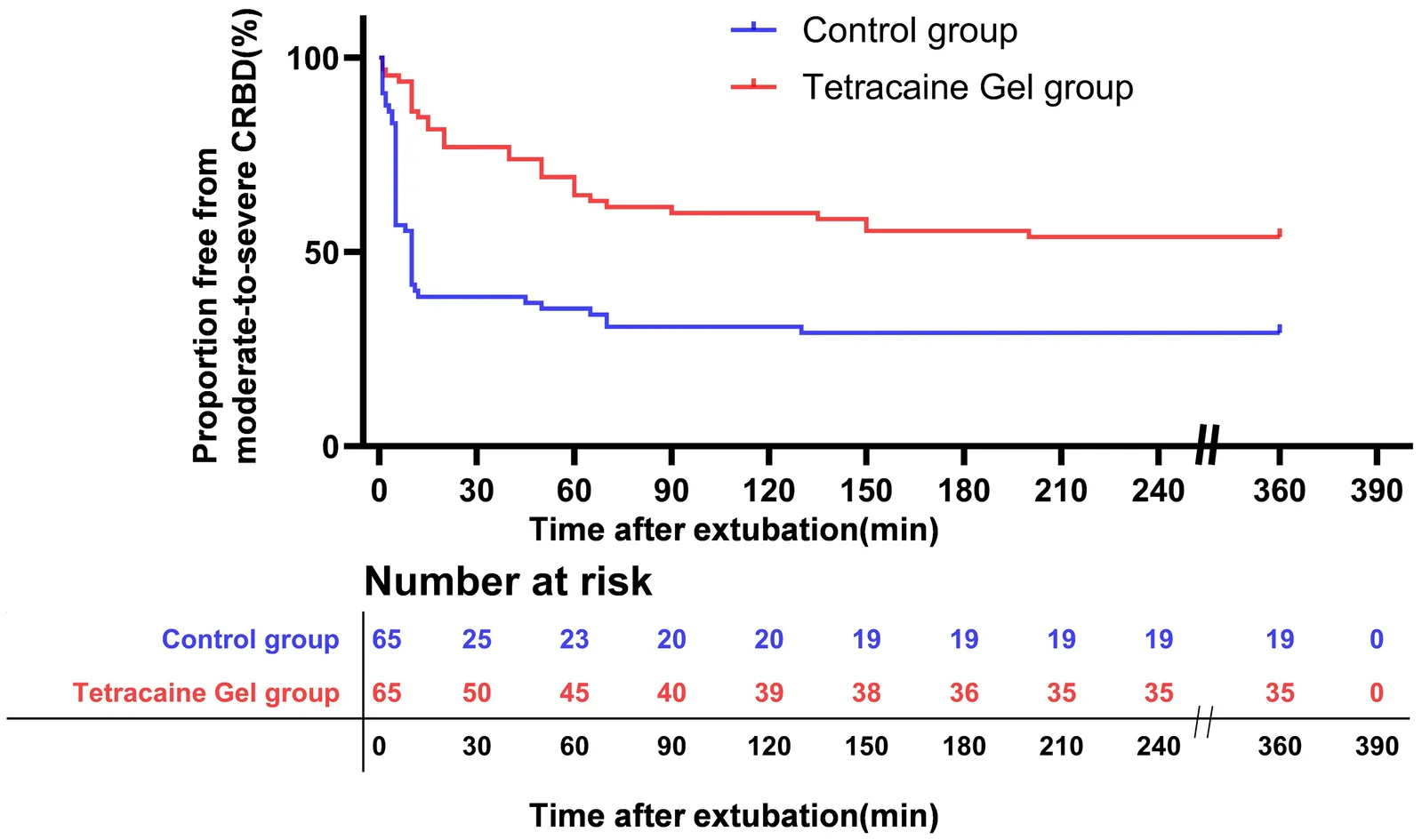
Kaplan–Meier analysis of the time to first-reported moderate-to-severe CRBD. Kaplan–Meier curves illustrate the proportion free from moderate-to-severe CRBD during the 360-minute observation period after extubation in the tetracaine gel group (red line) *versus* the control group (blue line). The event was defined as the first recorded occurrence of moderate-to-severe CRBD. Participants who had not developed moderate-to-severe CRBD by 360 minutes were censored at the 360-minute time point. The difference between the two groups was assessed using the log-rank test. The tetracaine gel group showed a significantly delayed onset of moderate-to-severe CRBD compared with the control group. Consistently, when the tetracaine gel group was used as the reference, the control group had a higher hazard of developing moderate-to-severe CRBD (HR for control *vs.* tetracaine gel, 2.299; 95% CI [1.453–3.637]; log-rank *χ*^2^=14.62; *p* < 0.001). CRBD, Catheter-related bladder discomfort; HR, Hazard ratio; CI, Confidence interval.

The overall incidence of moderate-to-severe CRBD within 24 h was significantly lower in the tetracaine gel group (30 [46.2%] *vs.* 46 [70.8%]; RR = 0.354, 95% CI [0.172–0.730]; *p* = 0.004), indicating a clinically important difference (Table 2). The incidence of new-onset moderate-to-severe CRBD within the first 0.5 h after extubation was significantly lower in the tetracaine gel group (15 [23.1%] *vs.* 43 [66.2%]; RR = 0.153, 95% CI [0.071–0.332]; *p* < 0.001) (Table 2 and Fig. 3). In contrast, during the 0.5–2 h period, the incidence was higher in the tetracaine gel group (11 [16.9%] *vs.* 2 [3.1%]; RR = 6.417, 95% CI [1.362–30.228]; *p* =0.016) (Table 2 and Fig. 3). No significant between-group differences were observed during the 2–4 h and 4–6 h periods (Table 2 and Fig. 3).

**Figure 3:**
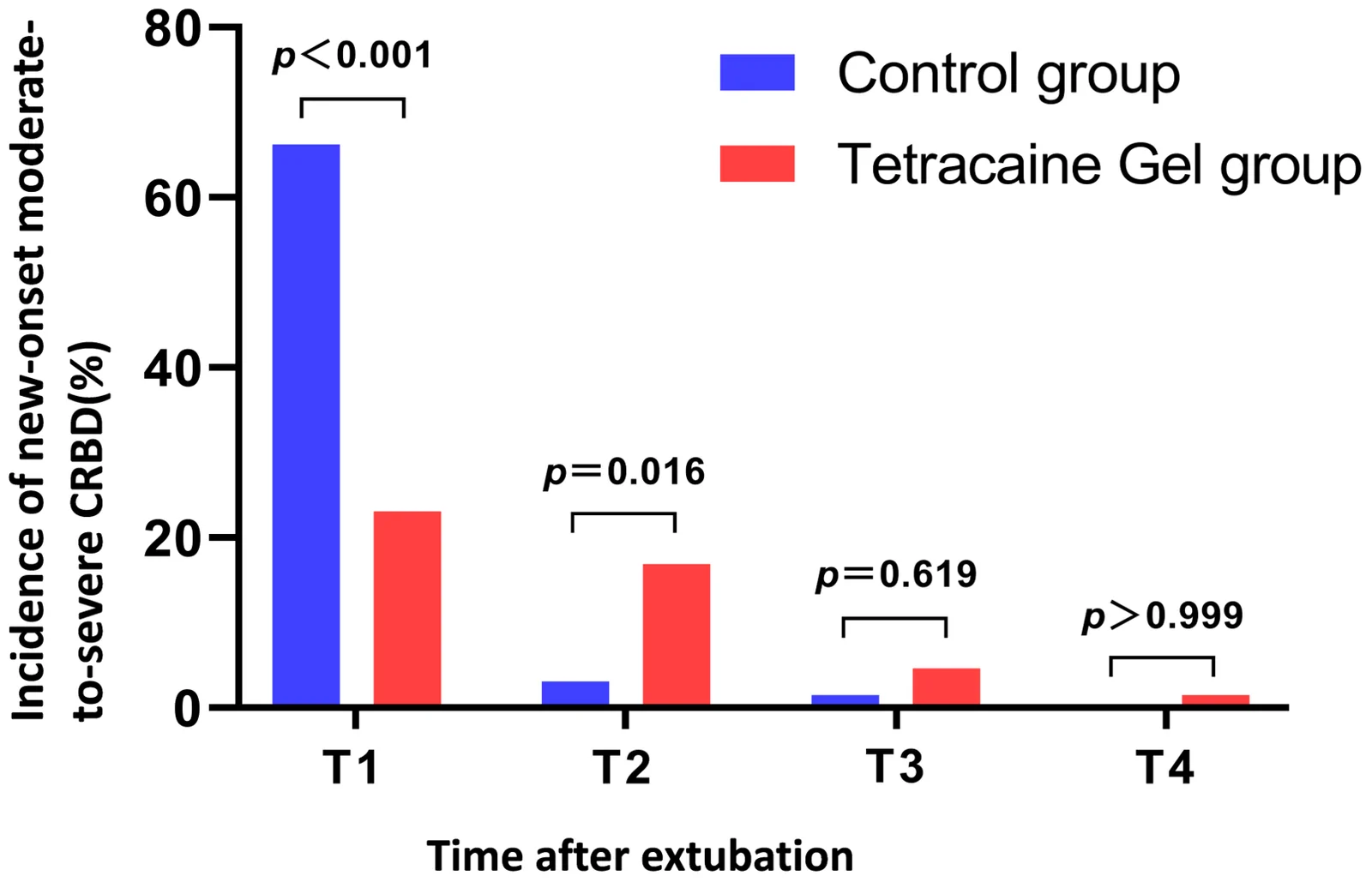
Incidence of new-onset moderate-to-severe CRBD during sequential time intervals after extubation. T1: 0–0.5 h after extubation; T2: 0.5–2 h after extubation; T3: 2–4 h after extubation; T4: 4–6 h after extubation. CRBD, Catheter-related bladder discomfort.

*post-hoc* subgroup analyses were performed using multivariable logistic regression models to explore the effects of tetracaine gel on moderate-to-severe CRBD within 6 h after extubation, stratified by age (<65 *vs.* ≥65 years) and procedure type (TURBT *vs.* TURP) (Table 3). In the overall population (*n* = 130), tetracaine gel significantly reduced the odds of moderate-to-severe CRBD compared with the control group (OR = 0.35, 95% CI [0.16–0.74], *P* = 0.006).

For age subgroups:

In patients aged <65 years (*n* = 39), tetracaine gel was associated with a significantly lower risk of moderate-to-severe CRBD (OR=0.24, 95% CI [0.06–0.92], *P* = 0.038). In patients aged ≥65 years (*n* = 91), the reduction in CRBD risk did not reach statistical significance (OR=0.43, 95% CI [0.17–1.06], *P* = 0.066). The interaction test for age subgroup was not statistically significant (*P* for interaction = 0.392).

For procedure type subgroups:

In patients undergoing TURP (*n* = 107), tetracaine gel significantly reduced the odds of moderate-to-severe CRBD (OR=0.34, 95% CI [0.15–0.79], *P* = 0.012). In patients undergoing TURBT (*n* = 23), the difference in CRBD risk between groups was not statistically significant (OR=0.39, 95% CI [0.07–2.39], *P* = 0.312). The interaction test for procedure type subgroup was not statistically significant (*P* for interaction=0.883).

Patient satisfaction scores at 6 h after extubation were significantly higher in the tetracaine gel group than in the control group (6.7 ± 0.5 *vs.* 6.2 ± 0.8, *p* < 0.001). Postoperative pain scores were lower in the tetracaine gel group at all assessed time points: post-extubation 0.5 h (2 [2–3] *vs.* 3 [2–4], *p* < 0.001), 2 h (2 [2–2] *vs.* 2 [2–3], *p* =0.001), 4 h (1 [0–1] *vs.* 1 [1–2], *p* =0.003), and 6 h (0 [0–1] *vs.* 0 [0–1], *p* =0.034). In addition, the number of patients requiring rescue analgesia showed no significant intergroup differences (28 [43.1%] *vs.* 33 [50.8%], *p* =0.380). Moreover, no postoperative delirium or surgical site infections occurred in either group, and the length of hospital stay did not differ significantly between groups (5 [4–6] days *vs.* 5 [4–6] days, *p* =0.373) (Table 4).

**Table 3:** *Post-hoc* subgroup analysis of the effect of tetracaine gel on moderate-to-severe CRBD within 6 hours after extubation, stratified by age and procedure type.

Variables	*n* (%)	Control group	Tetracaine gel group	OR (95%CI)	*P*	*P* for interaction
All patients	130 (100.00)	46/65	30/65	0.35 (0.16∼0.74)	0.006	
Age						0.392
<65 years	39 (30.00)	12/17	8/22	0.24 (0.06∼0.92)	0.038	
≥65 years	91 (70.00)	34/48	22/43	0.43 (0.17∼1.06)	0.066	
Procedure type						0.883
TURBT	23 (17.69)	5/11	3/12	0.39 (0.07∼2.39)	0.312	
TURP	107 (82.31)	41/54	27/53	0.34 (0.15∼0.79)	0.012	

**Notes.**

Data are presented as *n* (%) for patient distribution, and the number of patients with moderate-to-severe CRBD/total patients in each group. OR and 95% CI were calculated using multivariable logistic regression models. *P* values for interaction tests were provided to assess whether the effect of tetracaine gel differed significantly across subgroups.

CRBD Catheter-related bladder discomfort OR Odds ratio CI Confidence interval TURBT Transurethral resection of bladder tumor TURP Transurethral resection of the prostate

**Table 4:** Postoperative recovery profiles and secondary outcomes.

Variable	Control Group (*n* = 65)	Tetracaine Gel Group (*n* = 65)	*p*-value
Patient satisfaction at 6 h	6.2 ± 0.8	6.7 ± 0.5	<0.001
Postoperative pain score (NRS)			
At 0.5 h	3 [2–4]	2 [2–3]	<0.001
At 2 h	2 [2–3]	2 [2–2]	0.001
At 4 h	1 [1–2]	1 [0–1]	0.003
At 6 h	0 [0–1]	0 [0–1]	0.034
Postoperative analgesic requirement within 24 h	33 (50.8%)	28 (43.1%)	0.380
Postoperative delirium within 24 h	0 (0.0%)	0 (0.0%)	1
Surgical site infection	0 (0.0%)	0 (0.0%)	1
Length of hospital stay, days	5 [4–6]	5 [4–6]	0.373

**Notes.**

Data are expressed as means ± standard deviation, medians [interquartile range], or number (%). Postoperative analgesic requirement includes the use of any rescue sufentanil, tramadol, dezocine or non-steroidal anti-inflammatory drug within the first 24 h after surgery.

NRS Numerical rating scale

## Discussion

In our study, the application of tetracaine gel to the urinary catheter significantly delayed the time to first-reported moderate-to-severe CRBD. This delay was most evident within the immediate post-extubation period (0–30 min), during which both the incidence and severity of CRBD were markedly reduced. Notably, a transient increase in CRBD incidence was observed in the tetracaine group during the subsequent 30–120 min window, suggesting a time-limited anesthetic effect. Patient satisfaction was significantly improved with tetracaine gel, and postoperative pain scores were lower at all assessed time points, without an increase in analgesic requirements or adverse events such as postoperative delirium or surgical site infections.

The primary mechanism by which tetracaine gel likely attenuates early CRBD is through topical anesthetic action on urethral and bladder mucosal sensory nerves. Tetracaine, a long-acting ester-type local anesthetic, blocks sodium channels and inhibits the initiation and transmission of nociceptive signals from the catheterized urethra and bladder trigone (Zhao et al., 2024). This local effect may explain the marked reduction in CRBD incidence and severity observed in the first 30 min after extubation, a period during which catheter-related sensory stimulation is most acute. Our findings align with studies supporting the use of local anesthetics such as ropivacaine or bupivacaine for CRBD prevention, which similarly target peripheral sensory pathways (Veerman et al., 2021; Pournajafian et al., 2020; Lee et al., 2025; Binhas et al., 2011).

Furthermore, the incidence of CRBD following TURBT and other prostate-related urological surgeries (60–90%) is significantly higher than that after non-urological procedures (Abo Zeid et al., 2026; Veerman et al., 2021; Binhas et al., 2011). The mechanism involves the activation of mechanosensitive afferent C-fibers in the bladder mucosa and smooth muscle by direct irritation from the indwelling catheter and surgical trauma at the urethro-vesical anastomosis. These afferent signals trigger the micturition reflex. Within this reflex, parasympathetic pathways release acetylcholine, which stimulates muscarinic receptors on detrusor smooth muscle, leading to involuntary bladder contractions and the sensation of CRBD  (Veerman et al., 2021). The parasympathetic postganglionic neurons involved are situated in the detrusor wall layer and the pelvic plexus (Yan et al., 2025), with their fibers coursing alongside the prostatic vascular pedicles at the posterolateral aspect of the prostate (Loufopoulos et al., 2026). Targeted periurethral infiltration of local anesthetic can thus block the residual neural pathways at the apex (Loufopoulos et al., 2026).

Supporting this anatomical rationale, studies have shown that local infiltration of anesthetics around the bladder and urethro-vesical anastomosis reduces CRBD after radical prostatectomy, whereas dorsal penile nerve block alone does not confer the same benefit (Veerman et al., 2021; Weinberg et al., 2014). These findings underscore that blocking perivesical and periurethral neural transmission effectively inhibits the afferent limb of the CRBD pathway. In our study, tetracaine gel was applied uniformly over the entire catheter surface. During insertion, the gel provides localized anesthesia to the urethral mucosa, the prostatic fossa (in relevant cases), and the bladder neck/urethro-vesical junction. This direct topical blockade likely attenuates the afferent signals generated by catheter-induced bladder irritation, thereby reducing the incidence of postoperative CRBD.

The transient increase in CRBD incidence in the tetracaine gel group during the 30–120 min window warrants careful interpretation. This trend can be attributed to several factors: delayed symptom recognition as patients regain consciousness post-anesthesia, regression to the mean causing fluctuating rates in repeated measurements, and waning anesthetic effect of single-use tetracaine gel after the initial hour, which unmasks catheter-related sensory stimuli (Zhao et al., 2024). Additionally, prolonged nociceptive blockade may induce central sensitization and rebound hyperalgesia, a common phenomenon associated with local anesthetics, as the nervous system may upregulate excitatory signaling during the anesthetic period. Whether dose modification can relieve this effect remains to be determined, and relevant dose-finding studies should assess rebound pain and central sensitization markers. Nevertheless, the overall incidence within the full 360 min remained significantly lower in the tetracaine gel group, and Kaplan–Meier analysis confirmed a significantly longer time to first moderate-to-severe CRBD compared with controls. This delay may be clinically advantageous, as it shifts discomfort to a period when patients are more cooperative, thereby lowering the risk of agitated catheter removal during emergence.

The subgroup analyses revealed that the protective effect of tetracaine gel against moderate-to-severe CRBD was consistent in direction across different age and procedure type subgroups, although statistical significance was not achieved in all strata. All subgroup analyses in this study were exploratory, with interaction tests applied to evaluate potential effect modification. No significant interaction was observed, meaning the subgroup-specific findings should be interpreted cautiously and considered hypothesis-generating only, rather than evidence of true effect modification.

The apparent subgroup-level patterns may partly stem from differences in neural function among younger *versus* older patients, as well as variation in baseline CRBD risk and sample size across procedure subgroups. For instance, younger patients may have greater responsiveness to topical tetracaine gel owing to preserved neural function, whereas attenuated observable effects in older patients could relate to age-related neural degeneration, bladder dysfunction and polypharmacy (Hardy & Korstanje, 2023). The non-significant interaction test (*P* = 0.392) across age groups suggests that the between-age-group difference in statistical significance most likely reflects limited statistical power in the older stratum, rather than genuine differences in treatment efficacy. Likewise, the observed signal for benefit among patients undergoing TURP may correspond to their higher baseline CRBD risk. The non-significant result in the TURBT subgroup is plausibly driven by its small sample size (*n* = 23), and the non-significant interaction test (*P* = 0.883) provides no statistical support for effect modification by procedure type. Future studies with larger sample sizes in the TURBT population are warranted to confirm the efficacy of tetracaine gel in this specific group.

Of note, the tetracaine gel group yielded marginal improvements in postoperative pain scores and patient satisfaction, albeit reaching statistical significance. This should be discussed more critically. As reported previously (Veerman et al., 2021; Weinberg et al., 2014), local anesthetics targeting the bladder and urethra-vesical anastomosis can relieve CRBD but fail to substantially alleviate overall postoperative pain. This is because postoperative pain stems from multiple sources, including CRBD, prostatic resection trauma and urethral injury. While the urethral application of tetracaine gel provides topical analgesia at both catheter-irritation and surgical-trauma sites, the overall effect size remained small. This modest efficacy is likely explained by sufficient intraoperative analgesia, a short follow-up duration and the limited sample size of the present study. The absence of between-group differences in analgesic requirements, delirium, infection, or hospital stay further supports the safety profile of this intervention within the studied population.

Compared with systemic pharmacological approaches such as opioids, anticholinergics, or magnesium (Li et al., 2018; Zhou et al., 2021; Park et al., 2020), topical tetracaine offers a localized effect with minimal risk of systemic side effects. The ease of administration and the safety profile observed in this study support further evaluation of this approach as a potential option for routine use in patients undergoing urinary catheterization.

This study has several limitations. First, although postoperative assessors were blinded, the visible difference between tetracaine gel and the lubricant prevented complete blinding of the surgical team, potentially introducing performance bias. Second, despite preoperative education, some patients may have struggled to differentiate CRBD from incisional pain, which could affect symptom reporting accuracy. Third, various postoperative analgesics may alter patients’ subjective perception of CRBD and bias its reporting. We only documented cases of rescue analgesia but did not record drug types or dosages, so we could not adjust for confounding effects from different analgesic regimens. Fourth, as the findings of this study are exploratory and the difference in surgical methods may lead to variations in the outcomes, these findings need to be validated by larger-scale studies with more precise effect size estimation and longer follow-up.

## Conclusions

Under the conditions of this study, the topical application of tetracaine gel is a simple strategy to delay the onset and reduce the incidence of moderate-to-severe CRBD in the early postoperative period. Future studies should focus on determining the optimal dosing regimen, exploring strategies to prolong its efficacy, and validating the present findings in females, other catheterized populations and those receiving non-urologic surgeries. Meanwhile, subsequent investigations should standardize perioperative analgesic protocols and comprehensively record rescue drug specifications to enable adequate adjustment for analgesic confounders. Large-scale trials should stratify randomization by procedure type to explore efficacy across different urological conditions. Moreover, comparative studies between tetracaine and lidocaine—the commonly used topical anesthetic for urethral catheterization—are recommended to evaluate their efficacy and safety for CRBD prophylaxis. Beyond this, no major safety signal was observed in this sample, further pharmacological research is required to confirm safety and obtain data on pharmacokinetics and mucosal absorption.

## Supplemental Information

10.7717/peerj.21891/supp-1 Supplemental Information 1 CONSORT Checklist checklist of information to include when reporting a randomized trial

10.7717/peerj.21891/supp-2 Data S1 Raw data

10.7717/peerj.21891/supp-3 Supplemental Information 3 Raw data codebook

10.7717/peerj.21891/supp-4 Supplemental Information 4 Trial protocol

## Abbreviations

ASA: American society of anesthesiologists
BIS: Bispectral index
CRBD: Catheter-related bladder discomfort
PACU: Post-anesthesia care unit
TURBT: Transurethral resection of bladder tumor
TURP: Transurethral resection of the prostate

## Additional Information and Declarations

### Data Availability

The following information was supplied regarding data availability: The raw measurements are available in the Supplemental Files.

### Clinical Trial Registration

The following information was supplied regarding Clinical Trial registration: ChiCTR2500106854
