# Supplementary material for: Tetracaine hydrochloride gel delays and reduces catheter-related bladder discomfort after transurethral resection: a randomized controlled trial
Source: PeerJ. 2026 Nov 2;14:e21891. doi: 10.7717/peerj.21891 (PMC13738187; doi:10.7717/peerj.21891)
Supplement: Supplementary file 2 [file peerj-14-21891-s004.docx]

****The Effect of Tetracaine Gel on Catheter-Related Bladder Discomfort in Male Patients: A Randomized Controlled Study****

****Version:**** V2.0
****Version Date:**** 2025-06-01

****Protocol Summary:****

| ****Item**** | ****Description**** |  |
| --- | --- | --- |
| ****Project Title**** | The Effect of Tetracaine Gel on Catheter-Related Bladder Discomfort in Male Patients: A Randomized Controlled Study |  |
| ****Study Objective**** | This study aims to select patients scheduled for elective transurethral resection of bladder tumor or prostate surgery in our hospital. During catheterization, tetracaine gel will be used to observe the time to first complaint of moderate to severe CRBD within 24 hours postoperatively, the incidence of moderate to severe CRBD within 24 hours postoperatively, and the incidence of moderate to severe CRBD within 24 hours postoperatively in elderly (≥65 years) and young and middle-aged male (<65 years) patients. |  |
| ****Study Design**** | This is a single-center, prospective, randomized controlled study. |  |
| ****Total Sample Size**** | > 130 cases |  |
| ****Patient Selection**** | ****Inclusion Criteria:**** (1) Male, age > 18 years; (2) ASA classification I-III; (3) Elective transurethral resection of bladder tumor or prostate surgery. ****Exclusion Criteria:**** (1) Unstable hypertension, history of heart disease; (2) Severe liver or kidney dysfunction; (3) Mental illness or inability to cooperate and communicate; (4) History of allergy to local anesthetics; (5) Refusal to sign informed consent. |  |
| ****Treatment Plan**** | ****(1) Grouping and Intervention**** 130 patients will be randomly allocated into two groups (n=65 per group) using a random number table: ****Experimental Group:**** Before catheterization, tetracaine gel is evenly applied to the urinary catheter, which is then inserted. ****Control Group:**** Before catheterization, lubricating oil is evenly applied to the urinary catheter, which is then inserted.  ****(2) Anesthesia Method**** ****Preoperative Visit:**** Patients are visited the day before surgery and educated on how to identify CRBD (e.g., suprapubic discomfort or burning sensation with urinary urgency) and how to distinguish it from surgical pain.  ****Anesthesia Protocol:**** Routine vital signs are monitored. Anesthesia is induced with 0.05 mg/kg midazolam, 2 mg/kg of 1% propofol or 0.3 mg/kg etomidate, 0.6 mg/kg rocuronium or 0.15 mg/kg cisatracurium, and 0.5 ug/kg sufentanil. Anesthesia is maintained with 1 vol% sevoflurane, propofol (4-6 mg/kg/h), remifentanil (0.3 ug/kg/min), rocuronium (9-12 μg/kg/h) or cisatracurium (1-2 μg/kg/h). BIS is used for anesthesia depth monitoring. Before the end of surgery, the surgeon inserts the urinary catheter, inflates the balloon with 10ml of distilled water, and continuous infusion of saline for bladder irrigation is initiated. The patient is then transferred to the PACU for recovery. Residual muscle relaxation is reversed with sugammadex or neostigmine. The tracheal tube is removed when the patient's consciousness level (BIS > 90), awareness, and muscle strength have recovered. In the PACU, if the patient describes surgical site pain score ≥ 4, 1 μg/kg fentanyl is administered. If moderate or severe CRBD occurs and the postoperative pain score is ≥ 4, patients receive tramadol or fentanyl based on their predominant symptoms, followed by reassessment. |  |
| ****Efficacy Assessment**** | ****Effectiveness Evaluation Indicators (Primary and Secondary)**** ****Primary Outcome:**** Time to first complaint of moderate to severe CRBD within 24 hours postoperatively. ****Secondary Outcomes:**** Incidence of moderate to severe CRBD within 24 hours postoperatively; Incidence of moderate to severe CRBD within 24 hours postoperatively in elderly (≥65 years) and young/middle-aged male (<65 years) patients; Pain VAS score (at 2h, 6h, 12h, 24h); Incidence of delirium within 24 hours postoperatively (assessed using the Confusion Assessment Method, CAM); Analgesic requirement within 24 hours (calculated as equivalent fentanyl dose). ****Safety Evaluation Indicators:**** Tetracaine gel is a routinely used medication by anesthesiologists, and its safety profile is established. If related adverse events occur during the intervention, the intervention will be stopped immediately, and the effects will be observed. If severe tachycardia occurs, esmolol will be administered; if blood pressure is too high, nimodipine or sodium nitroprusside will be administered for antihypertensive treatment. |  |
| ****Statistical Methods**** | SPSS 22.0 statistical software will be used. The Kolmogorov-Smirnov test will assess the normal distribution of continuous data. Categorical variables will be analyzed using the Chi-square test or Fisher's exact test. Continuous variables will be analyzed using the independent samples t-test or Mann-Whitney U test. The time to first complaint of CRBD within 24 hours postoperatively will be analyzed using the t-test and Kaplan-Meier curves will be plotted. The Numeric Pain Rating Scale between the two groups at 4 different time points will be compared using the independent samples t-test, with repeated measures ANOVA used if necessary. Bonferroni correction will be applied, designating a P value of <0.0125 (0.05/4) as statistically significant for these comparisons. Post-hoc subgroup analysis will be used to assess the incidence of moderate or severe CRBD by age and CRBD grade. A P value <0.05 is considered statistically significant. |  |
| ****Study Duration**** | 6 months |  |

****I. Research Background:****

Catheterization is a routine perioperative procedure. However, after emergence from general anesthesia, patients with indwelling urinary catheters may experience urinary urgency or discomfort in the suprapubic region, which shares similarities with overactive bladder. This condition, primarily manifested as urinary urgency or burning sensation in the suprapubic area, frequency, urgency, and restlessness, is termed catheter-related bladder discomfort (CRBD) [1]. Especially in male patients, the incidence is 3.15 times higher than in female patients [2]. Previous studies have shown that the incidence of catheter-related bladder discomfort is high (47%-90%) and is a significant cause of emergence agitation in the post-anesthesia care unit (PACU), potentially leading to severe postoperative delirium. Male patients, due to their complex and narrow urethral anatomy, tolerate catheterization poorly and often experience more pronounced CRBD symptoms, which can induce stress responses like agitation, elevated blood pressure, and increased heart rate. This not only reduces patient comfort but may also prolong hospital stays, increase medical costs, and even affect postoperative recovery [3-4]. Therefore, CRBD is a significant issue requiring the attention and intervention of anesthesiologists.

Currently, the clinical management of CRBD primarily relies on systemic analgesics (e.g., opioids) or anticholinergic drugs. However, the former may cause systemic side effects such as respiratory depression, nausea, and vomiting, while the latter are limited by adverse reactions like dry mouth, constipation, and abnormal heart rate, posing higher risks, especially in elderly patients or those with underlying diseases [5]. Consequently, exploring direct intervention strategies with local anesthetics has become a research hotspot in recent years, aiming to alleviate CRBD symptoms at the source by inhibiting nerve impulse conduction in the urethra and bladder mucosa.

Tetracaine is an ester-type local anesthetic that inhibits nerve impulse conduction by blocking sodium channels, thereby reducing mucosal or skin sensation [6]. Theoretically, surface anesthesia of the urethral mucosa could block the transmission of nociceptive signals to the central nervous system, thus alleviating CRBD. Previous studies have shown that tetracaine gel can reduce the incidence and severity of CRBD in patients. However, the effect of tetracaine gel on the timing of CRBD relief, particularly the time to the patient's first complaint of CRBD, remains unstudied. Therefore, this study focuses on the male patient population, using tetracaine gel to observe the time to first report of CRBD, the incidence and severity of CRBD, and the occurrence of CRBD in patients stratified by age. This will provide a theoretical basis and clinical reference for the early use of preventive measures.

****II. Study Objective:****

This study aims to select patients scheduled for elective transurethral resection of bladder tumor or prostate surgery in our hospital. During catheterization, tetracaine gel will be used to observe the time to first complaint of moderate to severe CRBD within 24 hours postoperatively, the incidence of moderate to severe CRBD within 24 hours postoperatively, and the incidence of moderate to severe CRBD within 24 hours postoperatively in elderly (≥65 years) and young and middle-aged male (<65 years) patients.

****III. Study Design Type, Principles, and Procedures:****

****1. Study Design****
(1) ****Study Design Type:**** Single-center, prospective, single-blind, randomized controlled trial.
(2) ****Randomization Method:**** Patients will be numbered according to the order of inclusion and randomly assigned to the experimental group or the control group using a random number table. Group allocation will be placed in sealed, opaque envelopes, opened by an anesthesiologist not participating in this trial before surgery.
(3) ****Blinding:**** The anesthesiologist managing the intraoperative period and the researchers conducting postoperative follow-up will be blinded to the group assignment. The surgeon will be aware of the group assignment.
(4) ****Study Center:**** Jiangsu Provincial People's Hospital.
(5) ****Sample Size:**** 130 cases.

****2. Study Procedures****
(1) ****Grouping and Intervention****
130 patients will be randomly allocated into two groups (n=65 per group):
****Experimental Group:**** Before catheterization, tetracaine gel is evenly applied to the urinary catheter, which is then inserted.
****Control Group:**** Before catheterization, lubricating oil is evenly applied to the urinary catheter, which is then inserted.

(2) ****Anesthesia Method****
****Preoperative Visit:****
Patients are visited the day before surgery and educated on how to identify CRBD (e.g., suprapubic discomfort or burning sensation with urinary urgency) and how to distinguish it from surgical pain.

****Anesthesia Protocol:****
Routine vital signs are monitored. Anesthesia is induced with 0.05 mg/kg midazolam, 2 mg/kg of 1% propofol or 0.3 mg/kg etomidate, 0.6 mg/kg rocuronium or 0.15 mg/kg cisatracurium, and 0.5 ug/kg sufentanil. Anesthesia is maintained with 1 vol% sevoflurane, propofol (4-6 mg/kg/h), remifentanil (0.3 ug/kg/min), rocuronium (5-10 μg/kg/h) or cisatracurium (1-2 μg/kg/h). BIS is used for anesthesia depth monitoring.
Before the end of surgery, the surgeon inserts the urinary catheter, inflates the balloon with 10ml of distilled water, and continuous infusion of saline for bladder irrigation is initiated. The patient is then transferred to the PACU for recovery. Residual muscle relaxation is reversed with sugammadex or neostigmine. The tracheal tube is removed when the patient's consciousness level (BIS > 90), awareness, and muscle strength have recovered. In the PACU, if the patient describes surgical site pain score ≥ 4, 1 μg/kg fentanyl is administered. If moderate or severe CRBD occurs and the postoperative pain score is ≥ 4, patients receive tramadol or fentanyl based on their predominant symptoms, followed by reassessment.

****IV. Patient Selection:****

****1. Sample Size Calculation:**** Based on pilot study results and previous research findings, the time to first report of moderate to severe CRBD postoperatively for patients not using tetracaine gel is 0.2 hours (SD 0.5), and for patients using tetracaine gel, it is 2 hours (SD 5). Given 80% power and a 5% significance level, the sample size was determined to be 124, with 62 subjects per group using a 1:1 ratio. Given the anticipated low loss to follow-up rate in this study, we will recruit a final sample size of 130 patients (65 patients per group).

****2. Inclusion Criteria:**** (1) Age > 18 years; (2) ASA classification I-III; (3) Elective transurethral resection of bladder tumor or prostate surgery.

****3. Exclusion Criteria:**** (1) Unstable hypertension, history of heart disease; (2) Severe liver or kidney dysfunction; (3) Mental illness or inability to cooperate and communicate; (4) History of allergy to local anesthetics; (5) Refusal to sign informed consent.

****V. Research Methods and Technical Roadmap:****

[A flowchart would typically be placed here, describing the patient journey from enrollment, randomization, intervention, surgery, PACU, to postoperative follow-up at specified time points (2h, 6h, 12h, 24h) for outcome assessment.]

****VI. Outcome Measures:****

****Primary Endpoint:**** Time to first complaint of moderate to severe CRBD within 24 hours postoperatively.
****Secondary Endpoints:**** Incidence of moderate to severe CRBD within 24 hours postoperatively; Incidence of moderate to severe CRBD within 24 hours postoperatively in elderly (≥65 years) and young/middle-aged male (<65 years) patients; Pain VAS score (at 2h, 6h, 12h, 24h); Incidence of delirium within 24 hours postoperatively (assessed using the Confusion Assessment Method, CAM); Analgesic requirement within 24 hours (calculated as equivalent fentanyl dose).
****Other Indicators:**** Atropine and penehyclidine usage; Surgical time; Anesthesia time; Hospital stay length; Patient satisfaction (7-point Likert scale, 1 = very dissatisfied, 7 = very satisfied).

****Description of Primary Endpoint Measurement:**** Moderate to severe CRBD typically causes patients significant discomfort, prompting them to actively report it to doctors or nurses. Follow-up personnel will record this time.

****CRBD Severity Grading:****

****None:**** No discomfort.

****Mild:**** Urethral discomfort reported only when asked, tolerable.

****Moderate:**** Patient actively reports urinary urgency, dysuria, and suprapubic bloating, but without behavioral responses.

****Severe:**** Patient actively reports intense urinary urgency, dysuria, and suprapubic bloating, which is intolerable and accompanied by behavioral responses (e.g., severe restlessness, flailing limbs, attempting to pull out the catheter), requiring staff restraint and/or multiple caregivers.

****VII. Monitoring of Adverse Events:****

Tetracaine gel is a routinely used medication by anesthesiologists, and its safety profile is established. If related adverse events occur during the intervention, the intervention will be stopped immediately, and the effects will be observed. If severe tachycardia occurs, esmolol will be administered; if blood pressure is too high, nimodipine or sodium nitroprusside will be administered for antihypertensive treatment.

****VIII. Quality Control and Assurance:****

Researchers will strictly adhere to standard clinical trial processes to ensure the quality of this study.

****IX. Data Safety Monitoring:****

In this clinical study, senior physicians are responsible for quality control and data monitoring. Based on the potential risks during the perioperative period, the following data safety monitoring plan is established:

All adverse events will be recorded in detail, managed appropriately, and followed up until satisfactory resolution or stabilization. Serious adverse events and unexpected events will be reported promptly to the hospital's ethics committee, regulatory authorities, sponsor, and drug administration department as required.

Cumulative reviews of all adverse events will be conducted regularly every 2 months. If necessary, investigator meetings will be convened to assess the risk-benefit profile of the study.

****X. Statistical Analysis:****

SPSS 22.0 statistical software will be used. The Kolmogorov-Smirnov test will assess the normal distribution of continuous data. Categorical variables will be analyzed using the Chi-square test or Fisher's exact test. Continuous variables will be analyzed using the independent samples t-test or Mann-Whitney U test. The time to first complaint of CRBD within 24 hours postoperatively will be analyzed using the t-test and Kaplan-Meier curves will be plotted. The Numeric Pain Rating Scale between the two groups at 4 different time points will be compared using the independent samples t-test, with repeated measures ANOVA used if necessary. Bonferroni correction will be applied, designating a P value of <0.0125 (0.05/4) as statistically significant for these comparisons. Post-hoc subgroup analysis will be used to assess the incidence of moderate or severe CRBD by age and CRBD grade. A P value <0.05 is considered statistically significant.

****XI. Clinical Research Ethics:****

The clinical research will be conducted in accordance with relevant regulations, including the World Medical Association's Declaration of Helsinki. The trial protocol will be implemented only after approval from the Ethics Committee. Before enrolling a subject in this study, the investigator is responsible for providing a complete and comprehensive introduction to the purpose, procedures, and potential risks of the study to the subject or their legal representative. Written informed consent must be obtained. Subjects should be informed of their right to withdraw from the study at any time. The informed consent form should be retained as a clinical research document for reference. Subjects' personal privacy and data confidentiality will be protected throughout the research process.

****XII. Study Progress:****

August 2025 - December 2025: Clinical data collection

January 2026 - March 2026: Data collation and manuscript writing

****XIII. References:****

[1] Park JY, Baek JW, Yu J, Kim CS, Bae J, Kim YK. Vitamin C and catheter-related bladder discomfort after transurethral resection of bladder tumor: A double-blind, randomized, placebo-controlled study. J Clin Anesth. 2023 Oct;89:111191. doi: 10.1016/j.jclinane.2023.111191. Epub 2023 Jun 23. PMID: 37356194.
[2] Bach H, Kaasby K, Sørensen A, Løfqvist S, Laursen BS. Incidence and Severity of Catheter-Related Bladder Discomfort Among Nonurological Adult Patients in a Postanesthesia Care Unit. J Perianesth Nurs. 2020 Feb;35(1):29-33. doi: 10.1016/j.jopan.2019.06.013. Epub 2019 Oct 21. PMID: 31648873.
[3] Bai Y, Wang X, Li X, Pu C, Yuan H, Tang Y, et al. Management of catheter-related bladder discomfort in patients who underwent elective surgery. J Endourol 2015; 29:640--9. [https://doi.org/10.1089/end.2014.0670](https://doi.org/10.1089/end.2014.0670).
[4] Liang S, Pang Z, Zhou N, Liu Z, Guo Q, Huang J, Zou W. Development and validation of a prediction model for catheter-related bladder discomfort: a prospective observational study. Br J Anaesth. 2022 Dec;129(6):e147-e149. doi: 10.1016/j.bja.2022.08.018. Epub 2022 Sep 24. PMID: 36163078.
[5] Kim DH, Park JY, Yu J, Lee SA, Park S, Hwang JH, et al. Intravenous lidocaine for the prevention of postoperative catheter-related bladder discomfort in male patients undergoing transurethral resection of bladder tumors: a randomized, double-blind, controlled trial. Anesth Analg 2020;131:220--7. [https://doi.org/](https://doi.org/) 10.1213/ane.0000000000004405.
[6] Sun H, Mei Y, Zhu L, Sun Z, Yan J, Kikkawa DO, Lu W. Efficacy of 4% tetracaine gel and lidocaine-prilocaine cream in reducing local anesthetic injection pain in upper eyelid blepharoplasty: a randomized, single-blinded, controlled trial. BMC Ophthalmol. 2024 Dec 20;24(1):530. doi: 10.1186/s12886-024-03799-7. PMID: 39702190; PMCID: PMC11661032.
